# Retrospective Study of Perpetrators of Workplace Violence in a Large Urban Emergency Department in the United States

**DOI:** 10.3390/healthcare14030337

**Published:** 2026-01-29

**Authors:** Marla C. Doehring, Megan Palmer, Bruck Mulat, Marilyn Ives, Ashley Satorius, Andrew Beckman, Tabitha Vaughn, Benton R. Hunter

**Affiliations:** 1 Department of Emergency Medicine, Indiana University School of Medicine, Indianapolis, IN 46202, USAaesatori@iu.edu (A.S.); brhunter@iu.edu (B.R.H.); 2 Faculty Affairs and Professional Development, Indiana University School of Medicine, Indianapolis, IN 46202, USA; bmulat@iu.edu; 3 Michael and Susan Smith Emergency Department, Sidney and Lois Eskenazi Hospital, Indianapolis, IN 46202, USA

**Keywords:** workplace violence, patient safety, well-being, psychosocial risks, nursing, physician

## Abstract

**Background/Objectives:** Data on the perpetrators of workplace violence (WPV) in healthcare settings are lacking. We sought to identify characteristics of perpetrators of WPV in a United States emergency department (ED) and explore associations between patient demographics and acute visit features. **Methods:** This is a retrospective descriptive study of the perpetrators of WPV against ED healthcare workers (HCWs) identified in a previous prospective study. Perpetrator demographics and visit features are described. Regression analyses were performed to assess for associations between perpetrator demographics and visit features with physical violence (PV) and restraint use. **Results:** 91 WPV encounters were included. The average age was 44.8 years. Most patients (*n* = 48; 53%) did not have an active psychiatric complaint and were not intoxicated, but 71 (78%) had a history of psychiatric illness. Twenty-four events (26%) involved PV, which was more common among patients on an emergency detention (RR 2.18; 95% CI 1.12–4.23) but was not associated with any patient demographics after adjustment. Restraints were ordered in 33 (36%) patients. Age, sex, PV, and intoxication or active psychiatric complaints were associated with restraint use, but in adjusted analysis, only PV (RR 1.89; 95% CI 1.13–3.16) and active psychiatric complaint or intoxication (RR 2.26; 95% CI 1.21–4.22) remained associated with restraint use. **Conclusions:** Half of perpetrators in this study were neither intoxicated nor had an active psychiatric complaint. PV was more common among patients on emergency detention. Restraint use was more likely in PV events and patients who were intoxicated or had psychiatric complaints.

## 1. Introduction

The United States (U.S.) Occupational Safety and Health Administration defines workplace violence (WPV) as “any act or threat of physical violence, harassment, intimidation, or other threatening behavior that occurs at the work site. It ranges from threats and verbal abuse to physical assaults” [1]. WPV against healthcare workers (HCWs) is common worldwide, especially in the emergency department (ED) [2,3]. This is likely due to the stressful, unpredictable ED environment that may include long wait times, overcrowding, and patients who are experiencing acute mental health crises or are under the influence of drugs or alcohol.

An analysis of literature on WPV in the ED found it to be a global issue with publications from 50 countries with the U.S. (121), Australia (48), China (33), and Turkey (21) producing the most articles [2]. A recent study found that HCWs in a large ED in the U.S. experienced WPV once every 3.7 shifts [3]. According to the American College of Emergency Physicians, 79% of emergency physicians report experiencing or witnessing WPV, with 30% saying they experienced physical violence (PV) 2–5 times per year [4]. In addition, WPV negatively impacts HCWs with one recent study of emergency nurses citing WPV as decreasing their job satisfaction and contributing to their intention to leave their jobs [5]. Losing highly skilled and highly trained HCWs can jeopardize patient safety and the quality of patient care in the ED.

Although verbal abuse (threats, slurs, or vulgar insults) is the most prevalent form of WPV, PV makes up an important minority of WPV events, ranging from 9% to 43% of incidents reported [6,7]. In a meta-analysis of 26 ED-based studies, Aljohani et al. found that 72% of WPV cases involved verbal violence only, 18% involved PV, and 9.5% were other types of WPV (e.g., stalking, sexual harassment) [8].

Although previous studies have focused on collecting data about the incidence and prevalence of WPV, less is known about the characteristics of the perpetrators. This is especially true for studies based in the U.S. Previous studies have demonstrated that patients affected by active psychiatric complaints or substance use are more likely to be aggressors in WPV [9,10]. Perpetrators of WPV are often found to be young to middle-aged [11,12,13].

Often, patients exhibiting aggressive behavior are chemically sedated and/or physically restrained by ED staff or security. In one study, out of 83 incidents, twenty patients (24.1%) were sedated, and 16 (19.3%) were physically restrained [9]. The use of restraints may be associated with the severity of patient violence and the requirement for an immediate intervention to deescalate the incident.

The primary objective of this study was to describe the characteristics of ED patients in a large U.S. ED who were perpetrators of WPV. The secondary goals were to explore associations between perpetrator demographics and acute visit features with (1) WPV events that involved PV rather than verbal abuse only, or (2) the use of physical or chemical restraints. Having a better understanding of the situational and demographic characteristics of patient perpetrators can inform more targeted interventions to reduce WPV occurring in healthcare settings.

## 2. Methods

This is a sub-study of a previously published prospective study conducted over 2 months in 2023 exploring the frequency of WPV perpetrated against HCWs in the ED [3]. The study took place in the ED of an urban academic teaching hospital in the U.S. with an annual ED volume of approximately 100,000, which is approximately 50% male, 40% black, and 36% white. This site was selected for its high patient volume and diverse population, offering an opportunity to observe a wide range of clinical presentations within a single setting.

The methods of the parent study have been previously described [3]. Briefly, HCWs (faculty physicians, resident physicians, nurses, advanced practice providers, and other ED personnel) from a single, large urban ED in the U.S. were recruited to participate and informed consent was obtained from those HCWs. Those who agreed were asked to fill out a “shift sheet” at the end of each shift worked in the ED during the 2 month study period. The shift sheet asked whether they had experienced a WPV event during that shift, and, if so, to provide a brief description and rate the negative impact it had on them. Participants were asked to turn in a shift sheet after each shift, regardless of whether they had experienced a WPV event during that shift. After the study was completed, each event description was assessed by two authors and coded into 1 of 5 severity categories using a previously validated instrument [3,12,13]. Type 1 and 2 events are verbal abuse, with type 2 involving threats of violence or slurs against the HCW. Type 3 events involve PV such as punching, kicking, or spitting. Type 4 and 5 events are PV resulting in the need for medical attention (type 4) or permanent disability or death (type 5). The two authors coded each event, blinded to each other’s coding. Discrepancies were resolved through discussion, with initial agreement measured and reported as a kappa value.

For the current study, if an event had occurred, participants were asked to place a patient identification sticker on the shift sheet corresponding to the patient (or in some cases, the patient’s family member) who perpetrated the WPV event. This is a retrospective descriptive study of the patient perpetrators of WPV on HCWs identified prospectively in the parent study. Any patient with a sticker placed on one of the “shift sheets” as a WPV perpetrator was eligible for inclusion in the current study. There were no specific exclusion criteria, but if the same patient identifier was placed by multiple HCWs during the same visit, that was counted as a single patient and a single event. In the case of a single patient who perpetrated WPV on multiple different visits, each separate visit would count as its own event, so this was an event level analysis. Due to safety concerns for researchers, informed consent for participation from perpetrators of WPV was not required as per approval for waiver granted by the Indiana University Institutional Review Board (IRB Protocol # 19597).

Data on included patients was extracted from the electronic medical record by a trained medical student researcher using a standardized data extraction form. To ensure accuracy, 10% of charts underwent extraction by an additional trained author, and agreement between the two extractors was measured and reported. We collected demographic data (age, race, sex) as well as visit and patient level features, including whether the patient was noted to be intoxicated, have an active psychiatric complaint, have a history of psychiatric diagnosis, whether law enforcement was involved in the incident, whether physical or chemical restraints were ordered, and what the patient’s final ED disposition was. Intoxication was counted as “yes” if either there was mention of acute intoxication (alcohol or illicit drugs) in the provider or triage notes, or as a final diagnosis at patient disposition. A positive urine drug screen without mention of acute intoxication in the ED chart was not counted as intoxication. A history of psychiatric diagnosis was predefined as a documented history of any of the following: Alcohol or drug use disorder, antisocial personality, anxiety, bipolar disorder, borderline personality, conduct disorder, intermittent explosive disorder, oppositional defiant disorder, psychosis, schizoaffective disorder, or schizophrenia. Active psychiatric complaint was gathered from the visit chief complaint and provider notes, and included auditory or visual hallucinations, delusions, paranoia, suicidal ideation, homicidal or suicidal ideation or self-harm (see Appendix A for definitions and examples). For data analysis purposes, the time of ED check-in was divided into three timeframes: 7 a.m.–3 p.m. (first shift), 3 p.m.–11 p.m. (evening shift), and 11 p.m.–7 a.m. (night shift).

Demographic and patient level features were reported as counts (percentages) for categorical variables and as a mean with standard deviation (SD) for age. To assess features associated with PV and restraint use, we conducted univariate and multivariable analyses using a modified Poisson Regression with robust error variance. Results were reported as Relative Risks (RRs) with 95% confidence interval (CIs) in the univariate models, and as adjusted RR with 95% CIs in the multivariable models. Variables with potentially meaningful associations were eligible to be incorporated into the multivariable analyses. Since there were a small number of events (PV or restraint use) compared to the number of potential variables, we limited multivariable models to no more than 3 variables to be incorporated into the final models. Multiple models were explored, and final models were selected using information criteria (minimum Akaike information criterion/Bayesian information criterion). Model diagnostics included variance inflation factors (VIFs) for multicollinearity, the overall Wald test for joint significance of predictors, and goodness-of-fit using the normalized residual sum of squares (NRSS) test [14]. We report the final best-fit models for each outcome (PV and restraint use). All analyses were performed in Stata/SE 18 (StataCorp, College Station, TX, USA).

## 3. Results

**Figure 1:**
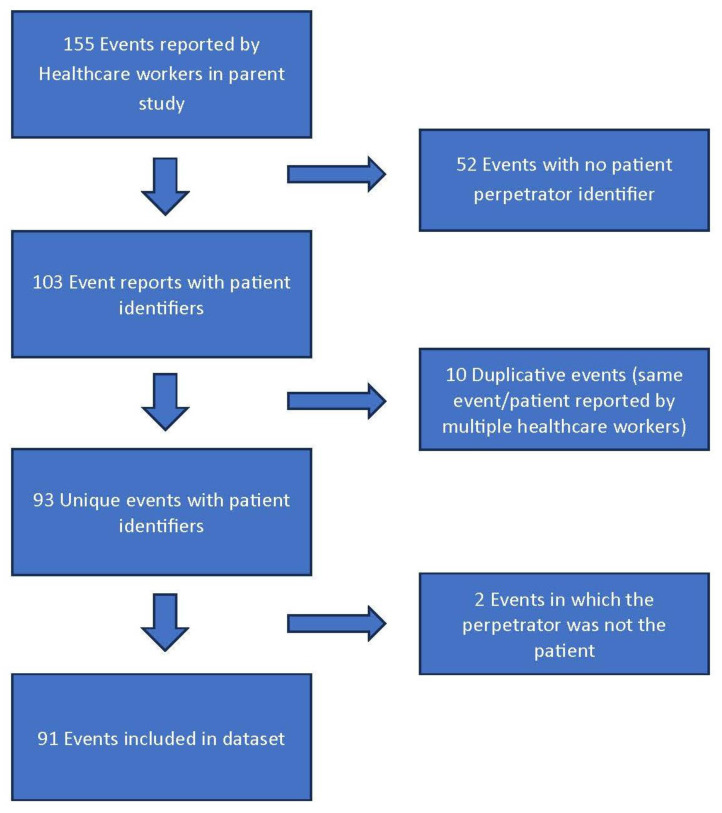
Flow of Study Patient Identification.

In total, 72 HCWs consented, participated in the original prospective study, and turned in 575 shift sheets over the 2-month study period. Of the 575 shifts with sheets turned in, there were 155 events reported for an average of 1 event for every 3.7 shifts. Of these events, 103 included a patient identifier. 10 events were excluded for being duplicative of the same event, and two were excluded because the offender was not a patient, so no demographic information was available. This resulted in 91 perpetrator-linked WPV events with identifiable perpetrators, which comprise the data set for the current study. Figure 1 demonstrates the flow of patient identification. Dual extraction was performed on 12 charts, with near perfect agreement (Kappa 0.88). Importantly, raw agreement was 100% for “intoxication (yes/no)” and 92% (1 disagreement out of 12) for “acute psychiatric complaint.”

Events that could be included in this study were similar to those that were excluded for lack of perpetrator identification. Specifically, victims were nurses in 72% vs. 75% and were 85% vs. 82% female. Physical violence occurred in 26% vs. 25%. Participants were asked to rate the impact the event had on them, and 24% in each cohort rated the impact as moderate or major (as opposed to minor or none).

For the primary outcome, demographics and descriptions of the patients are presented in Table 1. For context, the overall ED population is 50% male and 50% female, 40% black patients, and 36% white patients. As such, the population included in this study is generally representative of the overall patient population in these basic demographics. Of the 91 WPV events, the average patient age was 44.8 years. The patient was intoxicated in 34 (37%) events, had an active psychiatric complaint in 26 (29%), and had a history of psychiatric illness in 71 (78%). The slight majority of patients (*n* = 48; 53%) did not have an active psychiatric complaint and were not intoxicated. Law enforcement was present during 37 (41%) encounters; 17 (19%) patients had been placed under emergency detention, and 7 (8%) were under arrest at the time of their ED visit. Ultimately, 68 patients (75%) were discharged or left the ED, 16 (18%) were admitted with a medical or surgical condition and 7 (8%) were admitted by psychiatry.

Twenty-four events (26%) were coded as type 3 (PV). The remaining events were coded as types 1 or 2 (verbal abuse without PV). There were no type 4 or 5 events. Agreement between coders for the severity of events was near perfect (kappa 0.84). Table 2 displays the specific numerical results of univariate and multivariate analyses assessing for associations with PV. Given small cell counts and model instability, intoxication status and active psychiatric complaints were collapsed into a single indicator. This approach was supported by sensitivity analyses that yielded effect estimates of similar magnitudes and direction, along with improved model fit. In univariate analyses, the only explored variable associated with a statistically significant increased likelihood of PV was patients who had been placed on emergency detention by police or an emergency physician (RR 2.18; 95% CI 1.12–4.25). On average, patients involved in PV events were 3.4 years younger than patients involved in non-PV events (*p* = 0.41). PV was not associated with sex, race, intoxication, or active psychiatric complaints. The best fit multivariate model included check-in time, emergency detention, and active psychiatric complaint or intoxication. In this model, no variables were independently statistically significantly associated with PV. Check-in during the night shift showed a borderline but non-significant association with PV with an adjusted RR of 1.87 (95% CI 0.99 to 3.53). The NRSS test indicated no evidence of lack of fit (*p* = 0.924), no evidence of multicollinearity (all VIFs < 2.0), and the overall Wald test was significant ($\chi^{2}$(3) = 18.45, *p* < 0.001).

Table 3 shows the specific numerical results of analyses assessing for associations with the use of restraints, which were ordered in 33 (36%) patients. In univariate analyses, restraints were more likely to be used in younger patients (7.9 years younger on average; *p* = 0.05), those with acute psychiatric complaints OR intoxication (RR 2.57; 95% CI 1.38–4.780.), and type 3 events (RR 2.33; 95% CI 1.40–3.85). Restraint use was not associated with race, sex, or time of ED check in. The best fit multivariable model included type 3 event, sex, and active psychiatric complaint or intoxication. After adjustment for confounders, only type 3 event (adjusted RR 1.89; 95% CI 1.13–3.16) and active psychiatric complaint or intoxication (adjusted RR 2.26; 95% CI 1.21–4.22) remained associated with restraint use. The NRSS test indicated no evidence of lack of fit (*p* = 0.964), no evidence of multicollinearity (all VIFs < 2.0), and the overall Wald test was significant ($\chi^{2}$(3) = 26.51, *p* < 0.001).

## 4. Discussion

HCWs in the ED frequently experience WPV [3,4]. The ability to predict patient encounters with a higher likelihood of WPV could be valuable for determining the need for HCW caution or for dedicating resources, such as security or police presence. There is a paucity of literature from the U.S. describing perpetrators of ED WPV. In this dataset, we found that patients were most often the perpetrators of WPV (101 of 103 events reviewed), consistent with previous reports in the U.S. and Australia, while relatives or other patient surrogates are more commonly perpetrators in some other countries [6,12,15,16,17]. Consistent with other data from the U.S., this study found a slightly higher percentage of male perpetrators compared to females [13,16,18]. Studies outside the U.S. and Australia frequently report much higher percentages of male perpetrators [12,15,17]. Differing cultural norms and expectations may play a role in this discrepancy. While the average age of perpetrators in this study was 44.8 years, the range was 17 to 80, indicating that essentially any aged adult may commit WPV. The race demographics in our study were consistent with those of the study’s general ED population.

While WPV in the ED is often perpetrated by patients who have altered mental states due to acute intoxication or mental health crisis, more than half of the perpetrators in this study were neither intoxicated nor presenting with an active psychiatric complaint. The frequency of acute intoxication or mental health crisis in perpetrators of WPV is not well-studied, but perpetrators are often reported to be intoxicated or have psychiatric issues [13,19]. WPV perpetrated by sober patients who are not experiencing an acute mental health crisis may be more difficult to predict. Our data suggest that HCWs should not assume that patients without intoxication or active mental health complaints will necessarily be low risk for committing WPV. These events may be more disturbing to HCWs since there is no medical or psychiatric issue to attribute to the patient’s abusive behavior.

Among WPV events, those that involve PV are of special interest to HCWs and the ability to predict patients who are more likely to escalate to PV would be of significant value in creating safety plans and protecting vulnerable HCWs. Previous literature is scant regarding identifying patients at the highest risk of escalation to PV. We found that patients placed on an emergency detention were more likely to commit PV, but this association did not remain statistically significant after correction for other confounders. In the multivariable model, check in during the night shift (11 p.m.–7 a.m.) trended towards being predictive of an event resulting in PV with an adjusted RR of 1.87, although this was not quite statistically significant (95% CI = 0.99–3.53). Nonetheless, if confirmed in other studies, these results could suggest that appropriate caution and security presence should be emphasized during “night shifts,” when staffing may be more difficult. Increased WPV occurring during the night shift is supported by the findings of previous studies [9,20].

We also explored associations with the use of physical or chemical restraints. Unsurprisingly, events that involved PV (type 3 events) were most likely to be associated with restraint use in both univariate and multivariate analyses. This was found to be consistent with a study that identified an association between restraint use and increased patient PV [7]. Intoxication or active psychiatric complaints were also associated with increased likelihood, which is data that is supported by WPV literature [19,21]. Interestingly, females tended to be more commonly restrained than males, although this association was not statistically significant. Race was not associated with restraint use in this study, which may be different from some other studies in ED and prehospital settings where Black race has been associated with increased frequency of restraint use [22,23,24].

There are several limitations to this study. The assessment of WPV is subjective and thus vulnerable to individual perception and recall bias. However, the original study was prospective, and participants were asked to record their observations after each shift which limits recall bias. Of 155 events, we only had patient identifiers on 103, and it is not possible to determine whether the excluded events and the involved patients were similar to those with available information. For the event level variables that we had available to compare, which were mostly variables related to the victims of WPV rather than the perpetrators, the excluded events appeared nearly identical to those that were included. However, the possibility remains that there could be substantial differences between the perpetrators involved in events that were excluded and those included in this study. Timestamps for the exact time of each WPV event were not available so ED check-in time was used as an imperfect proxy. Since the WPV event may have occurred later during the ED stay, misclassifications in shift associations may have occurred. Coding the severity of violent events is not entirely objective, although all events were coded by at least 2 study personnel, with near perfect agreement. There is potential for chart abstractor bias due to the subjective nature of some records and reliance on medical records not designed for research purposes. However, in this study, many data points extracted were objective, and agreement was near perfect between the two team members who abstracted patient charts. With only 91 perpetrators in our dataset, the study is likely underpowered to detect clinically meaningful differences between subgroups. Further, with small numbers of outcomes (type 3 events, restraint use) in our Poisson models, we were limited in the number of variables that could be included in the multivariable models. Exact rates of acute intoxication and acute psychiatric complaints in all ED patients at the study site were not available. This makes interpretation of the frequency of intoxication and acute psychiatric illness more difficult; however, it is safe to say that our general ED population is well below the rates of 37% intoxication and 29% acute psychiatric illness we discovered among perpetrators. We were unable to address several potential confounders, such as certain social determinants of health like educational attainment and income level. These are areas for further future research. Finally, the study occurred in a single, large-volume, urban ED in the U.S. with 24/7 security presence, which may limit generalizability.

## 5. Conclusions

While WPV in the ED is often perpetrated by patients who have altered mental states due to acute intoxication or mental health crisis, about half of the perpetrators in this study were neither intoxicated nor presenting with an active psychiatric complaint. However, many of the perpetrators did have a history of psychiatric illness. Among patients involved in WPV events against HCWs, we found an increased likelihood of escalation to PV in patients who had been placed on emergency detention, although this association was not statistically significant after adjustment for other confounders. PV was not statistically significantly associated with age, sex, race, intoxication, or active psychiatric complaints. Physically violent events in this study more commonly occurred during the night shift, although this trend did not reach statistical significance. Further research addressing shift time differences in WPV is warranted. Restraints were more likely to be ordered for patients who were physically violent and those who were intoxicated or had psychiatric complaints. This study increases understanding of the situational and demographic characteristics of perpetrators of WPV and can inform more targeted interventions to reduce WPV occurring in the ED.

## Figures and Tables

**Table 1:** Demographics and Circumstances of the 91 Included Patients.

Mean Age (Standard Deviation); Range	44.8 Years (16.6); 17–80
Sex	40 Female (44%) 51 Male (56%)
Race/Ethnicity	44 Non-Hispanic Black (48%) 43 Non-Hispanic White (47%) 3 Hispanic (3%) 1 Unknown (1%)
Intoxicated	34 Yes (37%) 57 No (63%)
Active Psychiatric Complaint	26 Yes (29%) 65 No (71%)
History of Psychiatric Illness	71 Yes (78%) 20 No (22%)
Law Enforcement Present	37 Yes (41%) 54 No (59%)
Emergency Detention	17 Yes (19%) 74 No (81%)
Under Arrest	7 Yes (8%) 84 No (92%)
Final Disposition from ED	38 Discharged to Home (42%) 7 Discharged to Jail (8%) 9 Discharged After Psychiatric Evaluation (10%) 14 Left Before Being Discharged/AMA (15%) 16 Admitted Medically (18%) 7 Admitted to Psychiatry (8%)

ED = Emergency Department; AMA = Against Medical Advice.

**Table 2:** Associations with physical violence.

Variable	Univariate RR (95% CI)	Multivariable Adjusted RR (95% CI)
Active psychiatric complaint OR acutely intoxicated	1.86 (0.91 to 3.82)	1.67 (0.70 to 3.99)
ED check in during 11 p.m.–7 a.m.	1.67 (0.83 to 3.35)	1.87 (0.99 to 3.53)
ED arrival under emergency detention	2.18 (1.12 to 4.25)	1.81 (0.77 to 4.25)
Age (mean-centered, per 10 years)	0.91 (0.73 to 1.14)	N/A
Female (vs. Male)	1.28 (0.64 to 2.54)	N/A
Black Race (vs. white or other)	1.07 (0.54 to 2.13)	N/A

RR = Relative Risk; CI = Confidence Interval; ED = Emergency Department.

**Table 3:** Associations with Restraint Use.

Variable	Univariate RR (95% CI)	Multivariable Adjusted RR (95% CI)
Type 3 event (physical violence)	2.33 (1.40 to 3.85)	1.89 (1.13 to 3.16)
Active psychiatric complaint OR acutely intoxicated	2.57 (1.38 to 4.78)	2.26 (1.21 o 4.22)
Female (vs. Male)	1.73 (0.99 to 3.02)	1.62 (0.95 to 2.75)
ED check in during 11 p.m.–7 a.m.	0.90 (0.45 to 1.77)	N/A
ED arrival under emergency detention	2.82 (1.79 to 4.48)	N/A
Age (mean-centered, per 10 years)	0.82 (0.68 to 1.00)	N/A
Black Race (vs. white or other)	0.69 (0.39 to 1.22)	N/A

RR = Relative Risk; CI = Confidence Interval; ED = Emergency Department.

## Data Availability

The data presented in this study are available on request from the corresponding author due to restrictions regarding waiver of informed consent as described above.

## Appendix A

	**Intoxication**	**Active Psychiatric Complaint**	**History of Psychiatric Illness**
Definition	Intoxication was counted as “yes” if either there was mention of acute intoxication (alcohol or illicit drugs) in the provider or triage notes, or as a final diagnosis at patient disposition. A positive urine drug screen without mention of acute intoxication in the ED chart was not counted as intoxication.	Mention in either the visit chief complaint or provider notes of acute auditory or visual hallucinations, delusions, paranoia, suicidal ideation, homicidal or suicidal ideation or self-harm.	A documented history of any of the following: Alcohol or drug use disorder, antisocial personality, anxiety, bipolar disorder, borderline personality, conduct disorder, intermittent explosive disorder, oppositional defiant disorder, psychosis, schizoaffective disorder, or schizophrenia.
Example(s)	“Patient presents after drinking a fifth of vodka” “Brought by police after smoking spice, agitated”	“Presents with c/o hearing voices telling him to harm himself” “Pt confused, states he is a disciple of Jesus”	Past Medical History of Schizophrenia
